# Right Ventricular Sex Differences in Patients with Idiopathic Pulmonary Arterial Hypertension Characterised by Magnetic Resonance Imaging: Pair-Matched Case Controlled Study

**DOI:** 10.1371/journal.pone.0127415

**Published:** 2015-05-21

**Authors:** Andrew J. Swift, Dave Capener, Charlotte Hammerton, Steven M. Thomas, Charlie Elliot, Robin Condliffe, Jim M. Wild, David G. Kiely

**Affiliations:** 1 Department of Cardiovascular Science, University of Sheffield, Sheffield, United Kingdom; 2 INSIGNEO, Institute for In Silico Medicine, University of Sheffield, Sheffield, United Kingdom; 3 Radiology Department, Sheffield Teaching Hospitals NHS Foundation Trust, Sheffield, United Kingdom; 4 Sheffield Pulmonary Vascular Disease Unit, Royal Hallamshire Hospital, Sheffield Teaching Hospitals NHS Foundation Trust, Sheffield, United Kingdom; Vanderbilt University Medical Center, UNITED STATES

## Abstract

**Purpose:**

Sex differences exist in both the prevalence and survival of patients with idiopathic pulmonary arterial hypertension (IPAH). Men are less frequently affected by the condition but have worse outcome as compared to females. We sought to characterise the sex related differences in right ventricular remodelling in age matched male and female patients with IPAH using cardiac magnetic resonance imaging (MRI).

**Methods:**

A case controlled pair-matched study was conducted with patients matched by age and sex. Steady state free precession (SSFP) MRI of the heart was performed at 1.5T. Cardiac volume, function and mass measurements were corrected for age, sex and BSA according to reference data.

**Results:**

40 age and sex matched patients with IPAH were identified. The mean age was 57 (SD 17) in both male and female cohorts. Men had proportionally lower right ventricular (RV) ejection fraction, RV stroke volume and LV stroke volume than females, p=0.028, p=0.007 and p=0.013, respectively. However, there was no significant difference in RV mass or haemodynamic indices of mPAP and PVR between males and females.

**Conclusion:**

Male patients with IPAH have proportionally worse RV function despite similar afterload. We hypothesise that adaptive remodelling of the RV in response to increased afterload in IPAH is more effective in females.

## Introduction

Sex differences exist in both the prevalence and survival of patients with idiopathic pulmonary arterial hypertension (IPAH) [1, 2]. The prevalence of IPAH is typically higher in female patients, suggesting underlying sex specific influences on disease occurrence, yet female patients with PAH exhibit better survival compared with male patients despite similar management [3–5]. It has been postulated that the female myocardium exhibits superior adaptation to elevated afterload. In an animal model the female left ventricle has been shown to undergo concentric hypertrophy with preservation of systolic function in response to elevated afterload, whereas the hypertrophy of the male left ventricle was associated with deterioration in systolic function [6].

Cardiac magnetic resonance imaging (MRI) is considered the gold standard method of assessing biventricular mass, volume and function [7], allowing direct visualisation and quantitation of the changes to the right ventricle (RV) in patients with pulmonary arterial hypertension [8–10]. Poor outcome of males has been attributed to progressive deterioration in RV ejection fraction as derived from MRI in comparison to females with IPAH [11]. Possible theories include deterioration in RV ejection fraction due to inadequate hypertrophy of the myocardium or maladaptive hypertrophy that is insufficient to maintain systolic function.

In this work we sought to characterise the sex related differences in right ventricular mass and ejection fraction corrected for age, sex and BSA with MRI in treatment naïve age matched male and female patients with IPAH.

## Materials and Methods

### Patients

Consecutive patients with IPAH who underwent MRI and right heart catheterisation (RHC) within a 48-hour period between May 2009 to April 2013 were identified from a database of a large volume, nationally designated PH referral centre [12]. Patients were treatment naïve at the time of MRI. Male and female patients were selected into the study as age—matched pairs. Approval for this analysis of imaging techniques was granted by our institutional review board, ref c06/Q2308/8. Informed consent was waived for this study.

### MR image acquisition

MRI was performed on a GE HDx (GE Healthcare, Milwaukee, USA) whole body scanner at 1.5T using an 8 channel cardiac coil. Using a cardiac-gated, multi-slice, balanced SSFP sequence (20 frames per cardiac cycle, FOV = 48, matrix = 256 x 256, BW = 125 KHz/pixel, TR/TE = 3.7/1.6 ms) short axis cine images were acquired. A stack of images in the short axis plane with slice thickness of 8 mm (2mm inter-slice gap) or 10mm (0mm inter-slice gap) were acquired fully covering both ventricles from base to apex. End-systole was defined as the smallest chamber area on each slice and end-diastole was defined as the first cine phase of the R-wave triggered acquisition.

### Image analysis

Image analysis was performed prospectively on a GE Advantage Workstation 4.1 and the observer was blinded to the patient clinical information. RV and LV endocardial and epicardial surfaces were traced manually using the stack of short axis cine images, to obtain RV end-diastolic (RVEDV) and end-systolic (RVESV), and LV end-diastolic (LVEDV) and end-systolic volumes (LVESV). From end-diastolic volume and end-systolic volumes, RV and LV ejection fraction (RVEF and LVEF) and RV and LV stroke volume (RVSV and LVSV) were calculated. Cardiac volumes, but not RVEF and LVEF, were indexed for BSA. To calculate right ventricular mass the inter-ventricular septum was considered as part of the LV. RV myocardial volume was calculated by multiplying the area of the RV wall by the slice thickness on each short axis slice using Simpson’s method [13]. Trabeculations were considered to be part of the myocardial mass and were excluded from the blood pool. The sum total of myocardial slice volumes for the RV and the mean density of myocardium (1.05 g/cm^3^) then provide an estimate of RV mass. Ventricular mass index was defined as RV mass divided by LV mass, expressed as a ratio.

### RHC

RHC was performed with a 7.5 Fr balloon-tipped thermodilution catheter (Becton-Dickinson, USA). RHC was typically performed via the internal jugular vein using a Swan-Ganz catheter. IPAH was defined at RHC by a mean pulmonary arterial pressure (mPAP) ≥ 25 mmHg at rest and with a pulmonary capillary wedge pressure (PCWP) of ≤15 mmHg. Cardiac output (CO) was measured by thermodilution. Pulmonary vascular resistance (PVR) was determined as follows: PVR = (mPAP—PCWP)/CO.

### Statistics

Cardiac MRI volumetric measurements indexed for BSA were corrected for age and sex and presented as percentage (%) predicted, using methods as described previously [14]. To establish predicted values for corrected cardiac volumes, regression equations were generated for age and sex from healthy volunteers based on two previously published reference data sets Maceria [15, 16] and Kawut [17], for example, corrected RVEDVI (%) = RVEDVI/predicted RVEDVI x 100. Using mean volume measurements at each decade presented in the paper by Maceria et al [Maceria] regression equations were derived, these were applied to the patients in the present study to derive individualised predicted CMR values. Comparisons between male and female patients were analysed using the independent t-test for continuous data, the chi-square for categorical data. Intraclass correlation coefficient and Bland-Altman plot analysis was used to determine the level of agreement between estimated CMR data derived from Maceria versus Kawut population data. A second observer repeated the CMR RV measurements in 15 patients; reproducibility was assessed using intraclass correlation coefficient analysis.

The interval from diagnostic evaluation until all-cause mortality or census was considered as the follow-up period. The prognostic significance of sex was assessed using uni-variate Cox proportional hazards regression analysis and Kaplan-Meier analysis was also performed to determine the prognostic significance of sex. Statistical analysis was performed using SPSS 19 (SPSS, Chicago, Ill) and for presentation of the data GraphPad Prism 5.05 (GraphPad Software, San Diego, Calif) software was used. A p-value < 0.05 was considered statistically significant.

## Results

40 female and 40 male patients were identified from a cohort of 134 patients with IPAH. The mean age was 57 (SD 17) in males and 57 (SD 16) in the female patient cohorts. Table 1 presents the demographic, invasive haemodynamic and corrected cardiac MRI indices in age matched males and female patients with IPAH.

**Table 1:** Demographic, invasive haemodynamic and corrected MR indices in age matched males and female patients with IPAH.

	Males N = 40	Female N = 40	P-value
**Demographics**			
Age (yrs)	59 ± 17	59 ± 16	0.957
WHO functional class II	II (5), III (25) and IV (10)	II (8), III (26) and IV (6)	0.197
**RHC**			
mRAP (mmHg)	12 ± 5	10 ± 6	0.195
mPAP (mmHg)	53 ± 9	51 ± 13	0.524
PVRI (WU.m2)	17.4 ± 6.8	18.5 ± 9.1	0.553
PCWP (mmHg)	11 ± 3	10 ± 3	0.061
CI (L/min/m2)	2.6 ± 0.7	2.7 ± 1.1	0.646
SvO2 (%)	61.7 ± 8.2	63.9 ± 9.3	0.295
**CINE cardiac MR values**			
RV EDVI (ml/m^2^)	101 ± 25	99 ± 41	0.816
RV ESVI (ml/m^2^)	72 ± 25	64 ± 31	0.203
RV EF (%)	30 ± 13	37 ± 14	0.019
RV SVI (ml/m^2^)	29 ± 13	35 ± 20	0.085
RV mass index (g/m^2^)	45 ± 16	42 ± 20	0.396
VMI (ratio)	0.87 ± 0.29	0.86 ± 0.34	0.894
LV EDVI (ml/m^2^)	49 ± 13	46 ± 14	0.472
LV ESVI (ml/m^2^)	20 ± 8	16 ± 7	0.018
LV EF (%)	60 ± 12	66 ± 10	0.025
LV SVI (ml/m^2^)	29 ± 9	31 ± 11	0.409
**Corrected CINE cardiac MR** *			
RV EDV corrected	125 ± 31	141 ± 58	0.685
RV ESV corrected	271 ± 99	283 ± 150	0.127
RV EF corrected	44 ± 19	55 ± 21	0.028
RV SV corrected	54 ± 21	75 ± 43	0.007
RV mass corrected	140 ± 52	156 ± 73	0.250
LV EDV corrected	62 ± 16	65 ± 18	0.514
LV ESV corrected	77 ± 30	69 ± 28	0.186
LV EF corrected	90 ± 17	96 ± 15	0.064
LV SV corrected	54 ± 17	65 ± 22	0.013

MRI data are expressed as percentage predicted

WHO = world health organisation, mRAP = mean right atrial pressure, mPAP = mean pulmonary artery pressure, PCWP = pulmonary capillary wedge pressure, PVR = pulmonary vascular resistance, CO = cardiac output, Svo2 = mixed venous oxygen saturations, RV EDV = right ventricular end-diastolic volume, RV ESV = right ventricular end-systolic volume index, RV EF = right ventricular ejection fraction, VMI = ventricular mass index, LV EDV = left ventricular end-diastolic volume index, LV ESV = left ventricular end-systolic volume index, LVEF = left ventricular ejection fraction, LV SV = left ventricular stroke volume index.

* corrected for age, sex and BSA (% predicted) Maceria [15, 16].

**Fig 1:**
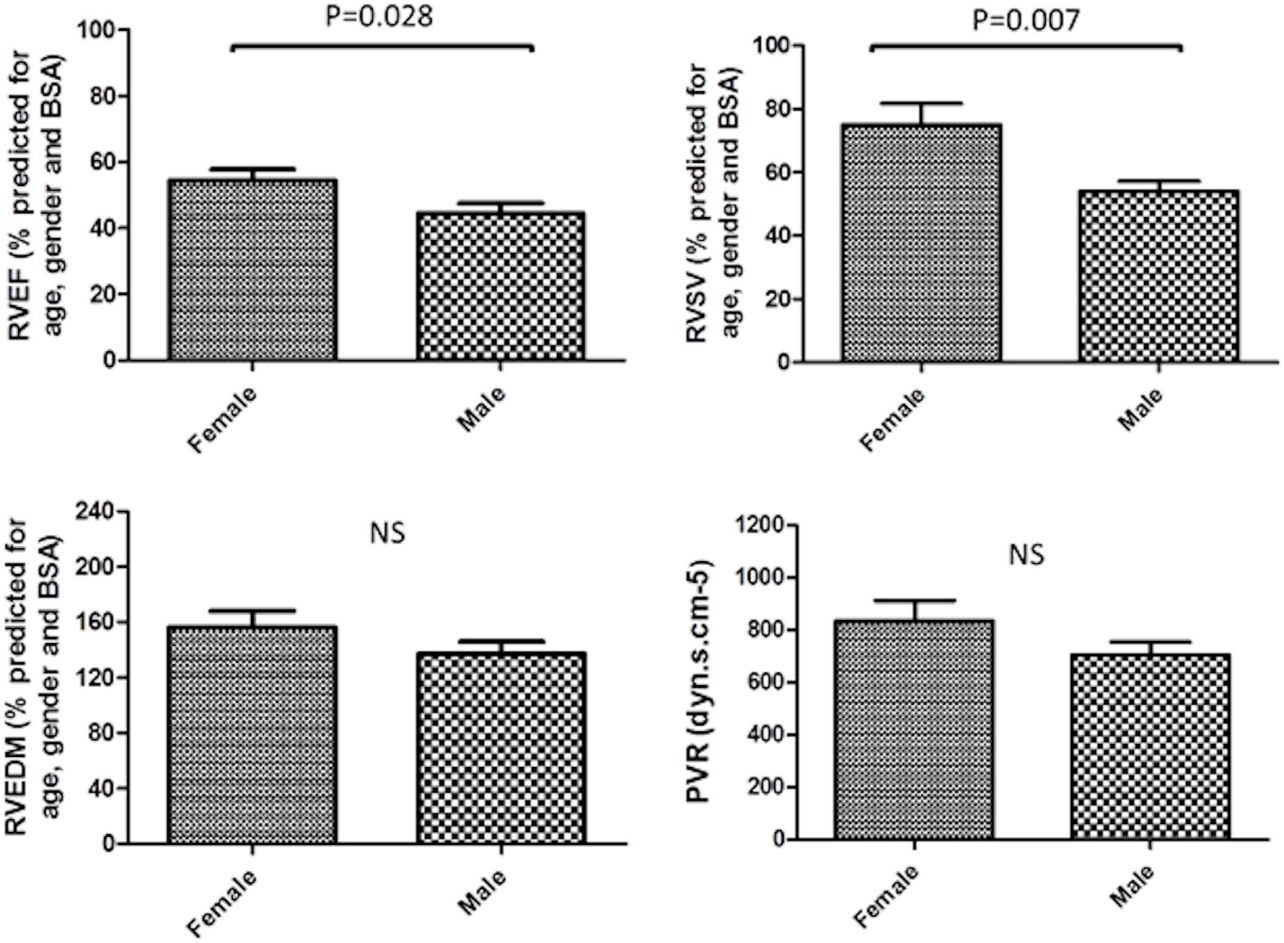
Bar charts showing reduced right ventricular ejection fraction (RVEF) and right ventricular stroke volume (RVESV) in male patients with IPAH. No significant (NS) difference between right ventricular end diastolic mass or pulmonary vascular resistance (PVR) between males and females. RVEF, RVSV and RVEDM corrected for age, sex (presented as % predicted)

Corrected for reference data, Maceira *et al*, men had proportionally lower corrected values of right ventricular (RV) ejection fraction and stroke volume, p = 0.028 and p = 0.007 respectively when compared to females, see Fig 1. In addition, LV stroke volume was lower in male versus female patients with IPAH, p = 0.013. Mean RV mass lower in males however this was not statistically significant (p = 0.250). Corrected for reference data, Kawut *et al* [17], RV ejection fraction was significantly lower in male patients than female patients, p = 0.045, whereas there was no significant difference in RV mass between males and females, p = 0.738, Table 2. Uncorrected RV and LV ejection fraction were lower and uncorrected LV end systolic volume was higher in male patients, there was no significant difference in uncorrected RV mass, RV end-diastolic or end-systolic volume or haemodynamic indices of mPAP and PVR and no significant difference in World Health Organisation functional class (WHO-FC) between males and females. In addition, VMI was not significantly different between males and females.

**Table 2:** Corrected RVEDV, RVEF and RV mass indices in age matched males and female patients with IPAH, correction using Kawut reference data[17].

	Males (N = 40)	Female (N = 40)	P value
**Corrected CINE cardiac MR** *			
RV EDV corrected	139 ± 36	164 ± 68†	0.041
RV EF corrected	40 ± 17	48 ± 18†	0.045
RV mass corrected	201 ± 80	223 ± 111	0.738

MRI data are expressed as percentage predicted.

BSA = body surface area, RV EDV = right ventricular end-diastolic volume, RV EF = right ventricular ejection fraction.

*corrected for age, sex and BSA (% predicted) Kawut [17].

Table 3 presents a comparison of pre and post cardiac volume mass and function between pre and post-menopausal women with IPAH. No significant difference was identified in RV volume, mass, WHO-FC or haemodynamic indices of mPAP and PVR between males and females. 11 men and 10 women were identified in a subgroup analysis of patients defined by the age criterion ≤50 years of age. Within this subgroup, male patients had lower mean corrected RV ejection fraction and RV stroke volume however this was not statistically significant.

**Table 3:** Comparison of cardiac volume mass and function between women above 50 (N = 30) and below or equal to the age of 50 (N = 10) with IPAH.

	Females ≤ 50yrs	Female >50 yrs	P-value
**CINE cardiac MR**			
RV EDVI (ml/m^2^)	102 ± 37	98 ± 42	0.776
RV ESVI (ml/m^2^)	71 ± 33	61 ± 31	0.434
RV EF (%)	31 ± 12	39 ± 15	0.148
RV SVI (ml/m^2^)	32 ± 15	36 ± 22	0.534
RV mass index (g/m^2^)	47 ± 24	40 ± 18	0.297
VMI (ratio)	0.85 ± 0.29	0.86 ± 0,32	0.862
LV EDVI (ml/m^2^)	53 ± 20	44 ± 11	0.083
LV ESVI (ml/m^2^)	18 ± 8	15 ± 6	0.346
LV EF (%)	67 ± 9	65 ± 10	0.596
LV SVI (ml/m^2^)	35 ± 15	29 ± 8	0.083
**Corrected CINE cardiac MR** *			
RV EDV corrected	129 ± 49	145 ± 61	0.440
RV ESV corrected	242 ± 117	297 ± 153	0.310
RV EF corrected	50 ± 18	56 ± 21	0.411
RV SV corrected	64 ± 30	78 ± 46	0.345
RV mass corrected	157 ± 81	156 ± 72	0.982
LV EDV corrected	67 ± 26	64 ± 16	0.583
LV ESV corrected	66 ± 29	69 ± 28	0.741
LV EF corrected	100 ± 12	95 ± 15	0.265
LV SV corrected	71 ± 31	65 ± 18	0.283

MRI data are expressed as percentage predicted.

RV EDV = right ventricular end-diastolic volume, RV ESV = right ventricular end-systolic volume index, RV EF = right ventricular ejection fraction, VMI = ventricular mass index, LV EDV = left ventricular end-diastolic volume index, LV ESV = left ventricular end-systolic volume index, LVEF = left ventricular ejection fraction, LV SV = left ventricular stroke volume index.

*Corrected for age, sex and BSA (%).

**Fig 2:**
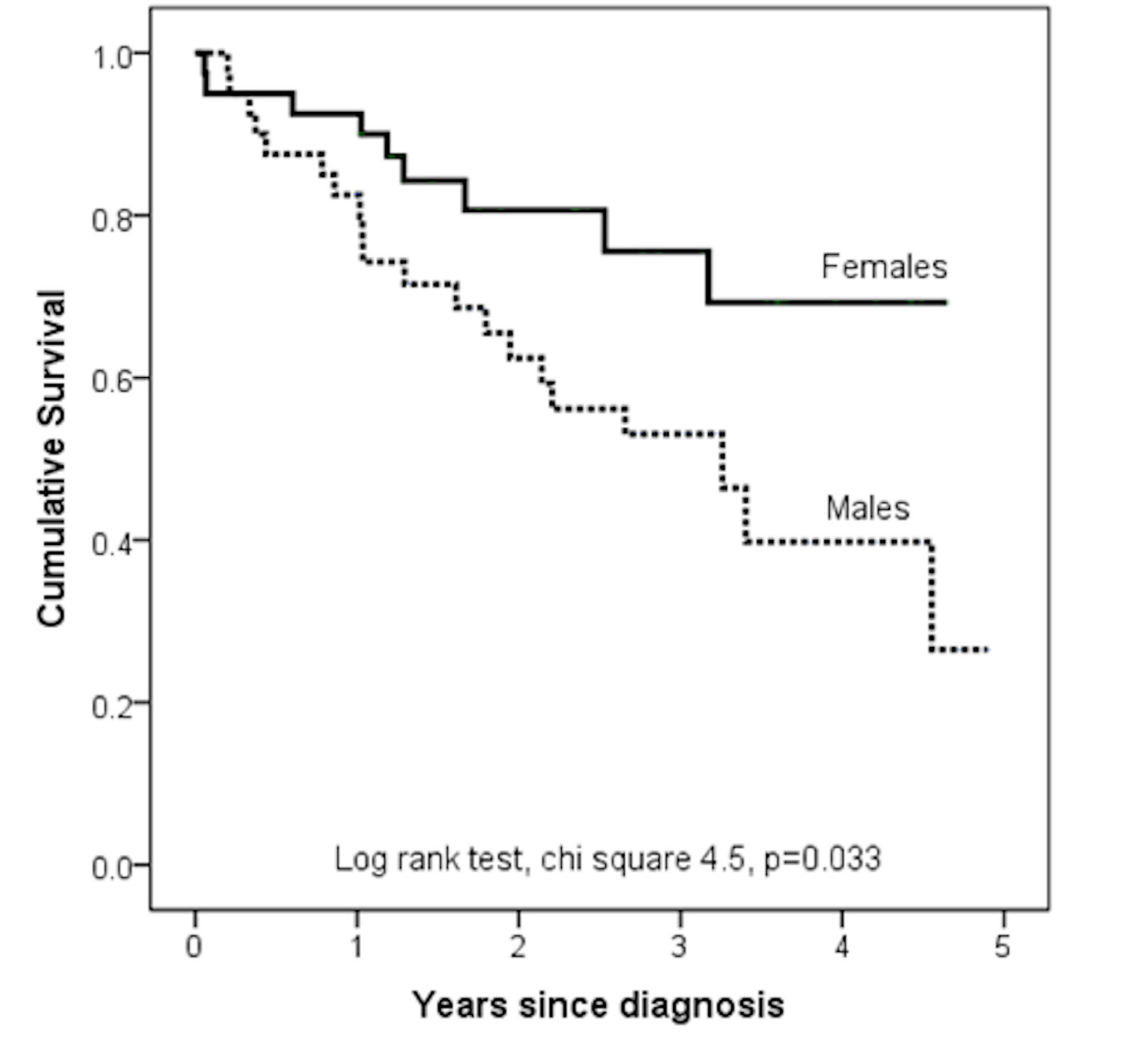
Kaplan Meier plot showing the prognostic significance of sex in age matched patients with IPAH.

There is a large systematic bias between Maceria et al and Kawut et al RV mass data, 34g +/- 9g higher by Maceria data. Estimated RV mass in our population for age, gender and BSA (Maceria) and height and weight (Kawut) correlate well (r = 0.917 p<0.0001). Only moderate agreement was identified at intraclass correlation coefficient analysis, 0.740 (CI 0.596 to 0.833). Estimated RV end diastolic volume for age, sex and BSA by Maceria and Kawut data show a strong correlation (r = 0.964, p<0.0001), and good agreement, intraclass correlation coefficient 0.981 (0.971 to 0.988). Estimated RV end-diastolic volume measured by Maceria et al data was 15ml +/- 9ml higher than by Kawut data. Right ventricular measurements had a good inter-observer reproducibility, with the intraclass correlation coefficient measuring 0.900–0.967. At Cox proportional hazards regression analysis male patients had significantly worse outcome than females Hazard Ratio 2.30 (1.05–5.08; p = 0.039), in addition males have higher mortality at Kaplan Meier analysis, p = 0.033, see Fig 2.

## Discussion

This study has shown that male treatment-naïve patients with IPAH have proportionally worse RV ejection fraction than females, despite having similar haemodynamic indices. This is the first study to compare cardiac MR indices in male and female patients with IPAH with correction of ventricular measurements for age, sex and BSA. We have shown that RV mass corrected for all three demographics tended to be lower in males, though was not significantly different between men and women. We postulate that the RV in men with IPAH does not adapt to elevated afterload as efficiently, and this may in part explain the worse outcome of male patients suffering from this condition.

In our study, uncorrected RV mass and end-diastolic volumes tended to be higher in male patient’s as is the trend in normal populations and as has been shown by Jacobs at al [11]. However, importantly when individual values are expressed as percentage predicted by age and sex, RV mass tended to be higher in females, suggesting that the RV of female patients with IPAH undergoes at least the same, if not more RV hypertrophy compared to males once correction has been made for age and sex. In addition, ventricular mass index a measure of RV hypertrophy corrected for LV mass which does not require correction for age and sex, was not significantly different between males and females further supporting the hypothesis that males and females undergo a similar level of RV hypertrophy in response to elevated afterload. RV volume was higher in male patients before correction as shown in previous studies [11, 15], following correction RV volume tended to be higher in females compared to males, this was a significant trend with correction using data from Kawut et al, but non-significant with correction using Maciera data. Whereas the trend for females to have better RV ejection fraction was significant regardless of the reference data used for correction and this difference was significant without correction of CMR data. In contrast to a study by Ventetuolo et al, [18] PVR tended to be higher in females, this difference was not statistically significant. Our cohort was made up of patients who were treatment naïve at the time of imaging, this may explain the difference from previous cohorts. Certainly in out cohort pulmonary hypertension was at least as severe in female patients as in male patients. We acknowledge that improved survival in female patients with PAH has been demonstrated in multiple registries as well as in specific cohorts, although not novel this study demonstrates the survival differences supporting previous studies [3, 11, 19, 20]. A recent study has investigated MRI derived RV measurements at baseline and follow-up in male and female patients with IPAH [11]. PVR and right ventricular ejection fraction were found to be similar at baseline in male and female patients and a similar reduction in PVR after one year was observed in both sexes. RV function, however, improved in female patients but deteriorated in male patients. The authors suggest that the RVEF response to therapy in patients with IPAH can to a large extent explain the worse survival seen in males suffering from the condition.

Gardener et al studied the sex differences in left venticular (LV) myocardial remodelling using an animal model [6]. In this study, female rats had lower mortality and minimal LV dilatation following fistula surgery, highlighting that sex differences exist in cardiac remodelling, with subsequent impact on the development of cardiac failure and patient outcome. The authors found the LV of female rats in response to increased afterload undergoes concentric hypertrophy capable of maintaining LV ejection fraction whereas the male LV underwent eccentric hypertrophy with less efficient contractility in response to elevated afterload, this mechanism may explain the sex differences found in our study.

It has been postulated that the sex differences may be the results of the protective effect of oestrogen. Animal studies in female and male mice have demonstrated acute vasodilator effects of oestrogen in vascular rings from both sexes and oestrogen level variations have been observed with menstruation, with less vasodilation found prior to ovulation at lower oestrogen levels [21]. In the present study no difference in cardiac MR characteristics was evident when women above and below 50 years of age were compared, however we note that simple classification of menopause by age is limited and further work looking at changes in the RV in well phenotyped pre and post-menopausal women is required.

Previous studies have suggested that female sex is associated with a lower prevalence and a better outcome of adult patients with heart failure [22, 23]. A population-based study investigated the implications of sex on the association between impaired LV function and mass. Myocardial adaptation to increased afterload differed between sexes with male subjects possessing a greater tendency to develop left ventricular dilatation and hypertrophy during the course of left ventricular dysfunction [23]. The authors postulated that the more favourable left ventricular remodelling associated with left ventricular dysfunction in women might have a role in disease progression and may ultimately contribute to better survival of women with heart failure. The present study shows that LV stroke volume is significantly better in female patients with IPAH, this measurement has been strongly linked to adverse outcome in patients with IPAH in previous studies, emphasising that it is not soley the changes in the RV that are important [14].

### Limitations

This is a single centre study. Failure to identify a significant difference in corrected RV mass between male and female patients could be due to type II error and should be validated at other centres. Relatively small numbers of patients in the less than 50 versus older than 50 female subgroups limits is a further potential source of type II error. Differences in the approach to segmentation of the ventricles between this study and others and whether trabeculations are included in right ventricular mass measurements and excluded from ventricular volume measurements is a potential source of error both for calculation of right ventricular mass and volume. Maceria et al, used a 3D segmentation approach as opposed to the manual multislice approach that we have used. Kawut et al’s method of cardiac segmentation was similar to ours, however the major difference is that Kawut et al included trabeculations in the blood pool whereas in this study and that of Maceria et al, RV trabeculations were considered to be part of the RV mass and therefore excluded from volume measurements.

There is a large difference in estimated RV mass in this study cohort when using regression equations derived from the two normal populations (Maceira et al and Kawut et al); the likely explanation is the inclusion of trabeculae in mass measurements by Maceria et al. Trabeculations make up a large proportion of the RV muscle mass and as such this difference is expected. The difference in RV end-diastolic is more surprising, Kawut et al find a smaller RV end-diastolic volume compared to Maceria et al, 15ml lower on average. Trabeculations were included in the RV volume measurements by Kawut et al, which would be expected to lead to a higher RV volume rather than lower. One explanation is that the 3D segmentation and tricuspid valve tracking method by Maceria et al may resulted in inclusion of a greater blood volume towards the base of the right ventricle. The second explanation is the relative small size of the study populations, limiting the applicability for use in other populations. However despite these observed differences in the scale of the variables, the trends with age and sex are similar between the two cohorts.

## Conclusions

This study indicates that there are sex differences in RV remodelling in patients with PAH. Despite males and females having a similar severity of disease, RV function was not preserved as effectively in response to elevated afterload.
